# Evaluating the efficacy of *Terminalia chebula* Retz. 5% cream compared to hydroquinone 2% cream in the treatment of melasma

**DOI:** 10.22038/AJP.2024.23932

**Published:** 2024

**Authors:** Amir Emad Kheirieh, Fariba Sharififar, Mehdi Ansari Dogaheh, Fatemeh Dabaghzadeh, Simin Shamsi Meymandi, Behnoush Bakhshoudeh

**Affiliations:** 1 *Pharmaceutics Research Center, Institute of Neuropharmacology, Kerman University of Medical Sciences, Kerman, Iran*; 2 *Herbal and Traditional Medicines Research Center, Kerman University of Medical Sciences, Kerman, Iran*; 3 *Department of Clinical Pharmacy, Faculty of Pharmacy, Kerman University of Medical Sciences, Kerman, Iran*; 4 *Pathology and Stem Cell Research Center, Kerman University of Medical Sciences, Kerman, Iran*; 5 *Cutaneous Leishmaniasis Research Center, Mashhad University of Medical Sciences, Mashhad, Iran*

**Keywords:** Cream, Hydroquinone, Melasma, Terminalia chebula Retz

## Abstract

**Objective:**

Melasma is a multifactorial, chronic, acquired skin disorder of hyperpigmentation. *Terminalia chebula *Retz.* (T. chebula) *has shown antioxidant, anti-inflammatory and tyrosinase enzyme inhibitory activities. So, the present study was designed to evaluate the efficacy of *T. chebula* 5% cream compared to hydroquinone 2% cream in treating patients with melasma.

**Materials and Methods:**

The formulation of *T. chebula *5% cream was prepared. The stability and release study of the cream were performed. In this randomized, controlled, triple-blind clinical trial, participants with facial melasma were randomly assigned to receive *T. chebula* 5% cream or hydroquinone 2% cream at bedtime for 12 weeks. Modified Melasma Area and Severity Index (mMASI) scores were recorded for all the participants at the baseline and 4, 8, and 12 weeks after initiating the study.

**Results:**

No statistically significant differences regarding mMASI scores were detected between *T. chebula* and hydroquinone groups at each time point. The reduction in mMASI scores was statistically significant (p<0.05) in *T. chebula *group 4, 8, and 12 weeks after initiating the study. However, it reached statistical significance (p<0.05) in hydroquinone group 8, and 12 weeks after the study initiation. The frequencies of side effects especially skin irritation were significantly (p<0.05) lower in *T. chebula* group.

**Conclusion:**

*T. chebula* 5% cream could be as effective as hydroquinone 2% cream in treating melasma with fewer side effects.

## Introduction

Melasma is a multifactorial, chronic, acquired skin disorder of hyperpigmentation characterized by brown macules and patches in the sun-exposed areas of the skin and predominantly affects women of childbearing age. Melasma can result in reducing quality of life, negative social impact and psychological distress. To treat melasma, a variety of topical therapies like depigmenting agents are used. Of them, topical hydroquinone inhibits tyrosinase and is the gold standard to treat melasma. But it still has frequent side effects such as erythema, irritation, pruritus, desquamation, and exogenous ochronosis (Chang et al., 2022; Grimes et al., 2019; McKesey et al., 2020). Despite numerous treatment agents available for melasma, research to find more efficacious and safer alternatives is ongoing. There is also growing interest in the potential use of herbal medicines as novel therapeutic options to treat melasma because of their safety (Grimes et al., 2019; Mpofana et al., 2022).

*Terminalia chebula *Retz. (*T. chebula*), known as myrobalan, is a traditional plant native to India and Southeast Asia that belongs to the genus *Terminalia* and family Combretaceae. The most important part of the tree is the fruit (Bulbul et al., 2022; Nigam et al., 2020) which is used orally and topically in traditional medicine. Daily dosage range of *T. chebula *is from 3 to 9 g. Also, LD50 (lethal dose, 50%) of its oral administration is more than 2000 mg/kg in rats (Jokar et al., 2016). The major bioactive constitutes of the plant are hydrolysable tannins such as chebulic acid, chebulinic acid and punicalagin, phenolic acids such as gallic acid and ellagic acid, flavonoids such as quercetin and ascorbic acid. Some studies previously showed that *T.* *chebula *has antidiabetic, nephroprotective, neuroprotective, cardiotonic, wound healing, anti-inflammatory, antispasmodic, antiaging, antimicrobial, antioxidant and tyrosinase inhibitory properties (Akhtar and Husain, 2019; Ansari et al., 2011; Bulbul et al., 2022; Nigam et al., 2020). A preliminary clinical study reported that *T. chebula* 5% cream could decrease the human skin melanin content (Akhtar et al., 2012).

*T. chebula* antioxidant, anti-inflammatory and tyrosinase enzyme inhibitory activities make it a good choice for treating melasma. So, the current study was designed to evaluate the efficacy of *T. chebula* 5% cream compared to that of hydroquinone 2% cream in treating patients with melasma.

## Materials

### Preparation of T. chebula extract

The dried fruit of* T. chebula* was purchased from the local market. It was scientifically approved in Herbal and Traditional Medicine Research Center (HTMRC), Kerman University of Medical Sciences, Kerman, Iran, and the voucher specimen (number: H112) was kept in the herbarium of HTMRC as a reference.The *T. chebula* dried fruit was crushed in a hand mortar, pulverized in an electric grinder, and then, sieved through a 35-mesh. The resultant powder was macerated for 72 hours in 80% v/v ethanol (maceration method) (Yakaew et al., 2016). The obtained extract was concentrated in the rotary evaporation apparatus (Heidolph GR-202, Japan) at 50°C and dried in an oven at less than 40°C. Until the next experiments, the dried extract was stored at -20°C. The dried extract of *T. chebula* dried fruit was around 11.4% w/w.

### Total phenolic content determination

The folic-Ciocalteu method was used to determine the total phenolic content of *T. chebula* extract (Yakaew et al., 2016). According to the slope of gallic acid calibration curve (Y = 0.0071X + 0.151 R2 = 0.9999), the total phenolic content of *T. chebula* dried extract was approximately 6.93 ± 0.12 mg gallic acid equivalent in 100 mg *T. chebula* dried extract.

### Formulation and characterization of T. chebula cream

Based on the half-maximal inhibitory concentration (IC50), the effect of kojic acid on inhibiting tyrosinase was 2.5 times more than that of *T. chebula* extract (Ansari et al., 2011)*.* Its efficacy in treating melasma was also comparable to that of hydroquinone (Chang et al., 2022). So, *T. chebula* 5% cream was formulated and clinically evaluated. Moreover, the safety of the plant 5% cream had been previously examined in human beings (Akhtar et al., 2012).

*T. chebula* 5% cream (water in oil (w/o) emulsion) was made from ingredients like acetyl alcohol, liquid paraffin, Vaseline and ethylenediaminetetraacetic acid (EDTA). Methylparaben and propylparaben as preservatives and Tween 80 and Span 80 as surfactants were also used in the formulation. *T. chebula* dried extract (5%) and other aqueous phase constituents were first dissolved in water, and then the aqueous phase was slowly added to the oil phase at a temperature of 70°C. The formulation was placed in ambient conditions after stirring and homogenizing (Akhtar et al., 2012). Hydroquinone 2% cream was also prepared by the same method in Pharmaceutical Research Center, Kerman University of Medical Sciences, Kerman, Iran.

### Absorbance - concentration curve of T. chebula dried extract

The λmax of *T. chebula* dried extract in water was found to be 373 nm using the spectrophotometer (Synergy HTX, USA). The absorbance of standard concentrations of *T. chebula* dried extract in water was measured at 373 nm, and the calibration curve of the absorbance versus concentration was plotted (Y = 0. 0.012x + 0. 0159, R2 = 0.9992). 

### Stability test

The physical stability of the prepared *T. chebula* cream was tested by placing it in three different environments: a refrigerator (2-8°C), an oven (40°C with 75 percent humidity), and a room (15-25°C). The color, appearance, odor, consistency and emulsion stability (phase separation) of the formulation were examined over 24 weeks. No change in the aforementioned properties was observed after 24 weeks at the refrigerator and room temperatures, and after 8 weeks at the oven temperature. To test the chemical stability of *T. chebula* cream in each of the above - stated environments, 1 g of the cream was dissolved in a 100 ml volumetric flask with hydroalcoholic solution (20% ethanol) and brought to the volume before being placed in a sonicator for 15 minutes. The absorption of the solutions was measured at 373 nm using the UV-spectrophotometer, and the amount of the active ingredient (*T. chebula *content) remaining in the cream was determined based on the absorbance - concentration curve of *T. chebula* dried extract. Although the amount of *T. chebula *was decreased in the cream during 24 weeks, more than 90% of *T. chebula *content was stable after 24 weeks in the three mentioned temperatures. Thus, the cream is chemically stable (Apriani et al., 2018). 

It should be noted that the physical and chemical stability were evaluated at the intervals of zero, one, two, eight, twelve and twenty-four weeks. 

### In vitro release of active ingredients

The release of *T. chebula* extract was evaluated using Franz diffusion cell. Half a gram of the formulation was placed in a donor compartment, and 1.5 ml sample was drawn out from the receptor compartment at 0, 0.5, 1, 1.5, 2, 2.5, 3, 4, 6, 8 and 24-hour intervals. The temperature was maintained at 37°C. The amount of active ingredients released was calculated by measuring the absorbance of the obtained samples at 373 nm using the UV-spectrophotometer (ANWAR et al., 2020). The amounts of the released *T. chebula* extract after 8 and 24 hours were 91.05±1.83% and 99.63±0.64%, respectively. 

### Clinical study

#### Study design and setting

This study was a randomized, controlled, triple-blind clinical trial concurrently conducted in the dermatology clinics of Torbat-e-Heydariyeh and Kerman, Iran from January 2020 to January 2021. 

### Ethical consideration

This study (98000359) was approved by the ethical committee of Kerman University of Medical Sciences bearing the ethical approval Code IR.KMU.REC.1398.254. Also, it was registered in Iranian Registry of Clinical Trial (IRCT20110310006026N10). Before the start of the study, all the participants signed a written consent form. Moreover, all the patients’ information was kept confidential.

### Participants

Female patients diagnosed with epidermal facial melasma were included in the study. Non-including criteria were endocrine disorders such as thyroid disorders and polycystic ovary syndrome, corticosteroid therapy during the previous three months, use of oral contraceptive pills during the previous three months, use of photosensitizing drugs, use of spironolactone and antiepileptic drugs, use of anti-hyperpigmentation medications during the previous 6 months, use of other therapeutic agents for melasma like niacinamide and licorice, pregnancy and lactation. Patients with hypersensitivity to hydroquinone or *T. chebula *were excluded from the study.

### Randomization and blinding

Eligible participants were randomly assigned to receive *T. chebula* 5% cream or hydroquinone 2% cream with a 1:1 allocation ratio at bedtime for 12 weeks. Block randomization with a block size of four was used. 

The packaging and labeling of both creams were the same. A person not involved in this trial packaged both creams in plastic tube containers, labeled them as A or B and generated allocation sequences. The dermatologists, participants and outcome assessor were blinded to the treatment assignment until the end of the study.

### Interventions

The participants were asked to wash their faces at night, apply a thin layer of the cream (*T. chebula* 5% cream or hydroquinone 2% cream) to their facial melasma lesions and then wash their face again in the morning for 12 weeks. In addition, they were recommended to apply sunscreen with the sun protection factors of 50 all over their faces in the daytime and reapplied sunscreen every 3 hours if they were exposed to the sun.

### Assessments

Demographic characteristics of the participants including age, education, history of oral contraceptive pills usage, number of pregnancies, chronic medical conditions including hypertension, stroke, heart disease, respiratory diseases and arthritis, family history of melasma, applying sunscreen, Fitzpatrick’s skin type and duration of melasma were recorded.

The Modified Melasma Area and Severity Index (mMASI) is a simple, reliable and valid tool that is used for measuring the outcome and severity of melasma. The total mMASI score is calculated by rating the darkness and area of involvement of 4 areas of the face (forehead, right cheek, left cheek, and chin), and then they are inserted into an equation. This index ranges from 0 to 24 and a higher score represents a greater severity of melasma (Rodrigues et al., 2016).

The dermatologists assessed and recorded the mMASI score for all the participants at the baseline and after 4, 8, and 12 weeks of initiating the study using Wood's Lamp. 

Also, the percentage of melasma lesions improvement between the baseline and the end of the treatment was evaluated and divided into four categories: 

1. Lack of improvement: a reduction of less than 25% in mMASI score. 

2. Relative improvement: a reduction of 25% to 50% in mMASI score. 

3. Significant improvement: a reduction of 51 to 75% in mMASI score. 

4. Complete improvement: a reduction of more than 75% in mMASI score (Shamsi Meymandi et al., 2016). 

Moreover, the side effects of *T. chebula* and hydroquinone creams were recorded.

### Statistical analysis

Due to the length of the study and the conditions of Covid-2019, this study was conducted as a pilot one with a sample size of 15 participants in each group (Whitehead et al., 2016). Considering a 20% loss to follow-up, the final sample was 18 ones in each group.

All the data was analyzed using Statistical Package for the Social Sciences (SPSS) version 26. The differences between quantitative variables were assessed using an independent samples *t*-test, paired sample *t*-test and Mann–Whitney U test while qualitative variables were evaluated using chi-square test and Fisher’s exact test. The changes in mMASI scores between *T. chebula* and hydroquinone groups during the study were measured by a mixed model analysis of variance. Statistically significant changes were defined as p-values less than 0.05.

## Results

**Figure 1:**
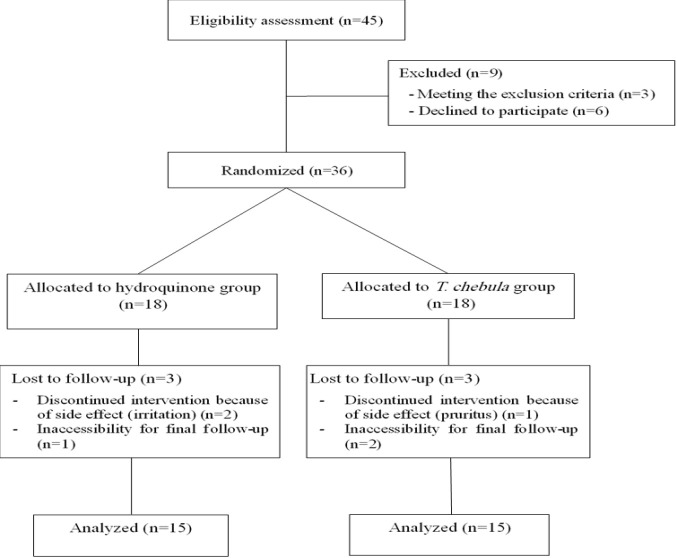
Flowchart of the trial

Out of the 36 randomized participants with melasma, 30 ones (15 in each group) completed the trial. Figure 1 shows the flowchart of the trial. The average age of the 30 participants was 22.73±6.98 years. The demographic characteristics of the participants in each group are presented in Table 1. There was not any statistically significant difference regarding these characteristics between the two groups. 

**Table 1:** Demographic characteristics of the participants in each group

**Variable**	***T. chebula*** ** group** **N (%)**	**Hydroquinone group** **N (%)**	**p-value** ^*^
Education			
High school Diploma Above of diploma	2 (13.33%) 12 (80.00%)1 (6.67%)	4 (27%)8 (53.33%)3 (20.00%)	0.423
Habitat			
City Town Village	2 (13.33%)9 (60.00%)4 (26.67%)	2 (13.33%) 5 (33.33%)8 (53.33%)	0.29
Family history of melasma			
Yes No	10 (66.67%)5 (33.33%)	13 (86.67%)2 (13.33%)	0.195
Fitzpatrick’s skin type			
Type 2 Type 3 Type 4Type 5	3 (20.00%)3 (20.00%)8 (53.33%)1 (6.67%)	2 (13.33%)8 (53.33%)5 (33.33%)0 (0.00%)	0.244
Applying sunscreen			
Always Rarely Never	0 (0.00%) 6 (40.00%) 9 (60.00%)	1 (6.67%) 2 (13.33%) 12 (80.00%)	0.18
Number of pregnancies			
0 1 2 3 4	5 (33.33%) 4 (26.67%) 3 (20.00%) 2 (13.33%) 1 (6.67%)	2 (13.33%) 2 (13.33%) 4 (26.67%) 5 (33.33%) 2 (13.33%)	0.446
History of oral contraceptive pills usage			
Yes No	11 (73.33%) 4 (26.67%)	11 (73.33%) 4 (26.67% )	0.654
Chronic medical conditions			
Yes No	1 (6.67%) 14 (93.33%)	2 (13.33%) 13 (86.67%)	0.5
Age (years) (Mean ± SD)	33.86 ± 7.67	35.6 ± 6.36	0.434^**^
Duration of melasma (months) (Mean ± SD)	6.64 ± 5.94	4.8 ± 4.26	0.726^**^

*T. chebula: Terminalia chebula *Retz., SD: Standard deviation. * Chi-square test or Fisher’s exact test. ** Independent samples* t-*test

The data was normally distributed based on the Shapiro–Wilk test. The analyses demonstrated that the group-by-time interaction (the difference between the groups at the different times) (p=0.259) and main effect of group (difference between the groups) (p=0.766) were not statistically significant for mMASI scores between *T. chebula* and hydroquinone groups after 12 weeks. But the effect of time (p=0.0001) (decrease in mMASI scores among all the participants regardless of grouping) reached statistical significance. Also, no statistically significant differences regarding mMASI scores were detected between *T. chebula* and hydroquinone groups at the baseline and after 4, 8 and 12 weeks of initiating the study. Table 2 presents the changes in mMASI scores between* T. chebula* and hydroquinone groups during the study.

The reduction in mMASI scores was statistically significant after 4, 8 and 12 weeks of initiating the study in *T. chebula *group. However, it reached statistical significance 8 and 12 weeks after the study initiation in hydroquinone group. The effect of each cream on reduction of mMASI scores between different time points is demonstrated in Table 3.

Two (13.33%) participants in the hydroquinone group and 5 (33.33%) ones in *T. chebula *group showed complete improvement. There was no significant difference between the two groups regarding improvement of melasma lesions at each time point (Table 4).

The side effects observed in the hydroquinone group were skin irritation (12 (80.00%) participants), desquamation (3 (20.00%) ones) and pruritus (2 (13.33%) ones). Also, irritation (2 (13.33%) participants) and pruritus (1 (6.67%) ones) were observed in *T. chebula *group. A significant difference was detected regarding irritation (p=0.001) between the two groups. But the difference did not reach statistical significance for desquamation (p=0.112) and pruritus (p=0.5). It should be mentioned that two participants in the hydroquinone group and one in *T. chebula *group respectively discontinued applying the cream because of irritation and pruritus.

**Table 2:** The changes in Modified Melasma Area and Severity Index scores between* T. chebula* and hydroquinone groups during the study

**Time point**	**Group**	**mMASI score (Mean±SD)** **(Median (IQR))**	**p-value** ^*^	**p-value** ^**^ **(time-group interaction)**
Baseline	*T. chebula*	5.43±2.27 4.80 (3.40-7.50)	0.512	0.259
	Hydroquinone	5.09±3.06 4.60 (2.40-7.20)		
After 4 weeks	*T. chebula*	4.37±1.72 4.80 (2.70-5.60)	0.806	
	Hydroquinone	4.36±2.37 3.60 (2.40-6.80)		
After 8 weeks	*T. chebula*	2.79±1.77 2.10 (1.40-4.60)	0.486	
	Hydroquinone	3.39±2.27 2.40 (1.80-4.80)		
After 12 weeks	*T. chebula*	2.1±2.55 1.80 (0.90-3.40)	0.233	
	Hydroquinone	2.7±1.76 2.40 (1.60-3.60)		

mMASI: Modified Melasma Area and Severity Index, *T. chebula: Terminalia chebula *Retz., SD: Standard deviation, IQR: Interquartile range (Q_1_-Q_3_).* Mann–Whitney U test. ** Mixed model analysis of variance

**Table 3:** Comparison of the effect of each cream on reduction of Modified Melasma Area and Severity Index scores between different time points

**Time point**	**Time point**	**p-value** ^*^	
		***T. chebula *** **cream**	**Hydroquinone cream**
Baseline	After 4 weeks	0.005	0.105
	After 8 weeks	0.0001	0.009
	After 12 weeks	0.0001	0.002
After 4 weeks	After 8 weeks	0.0001	0.001
	After 12 weeks	0.0001	0.0001
After 8 weeks	After 12 weeks	0.017	0.005

^*^Paired sample *t*-test

**Table 4:** Improvement of melasma lesions at each time point

**Time**	**Group**	**Complete improvement** **N (%)**	**Significant improvement** **N (%)**	**Relative improvement** **N (%)**	**Lack of improvement** **N (%)**	***p*** **-value** ^*^
After 4 weeks	*T. chebula*	0 (0.00%)	0 (0.00%)	6 (40.00%)	9 (60.00%)	0.333
	Hydroquinone	0 (0.00%)	1 (6.67%)	3 (20.00%)	11(73.33%)	
After 8 weeks	*T. chebula*	3 (20.00%)	3 (20.00%)	7 (46.67%)	2 (13.33%)	0.644
	Hydroquinone	1 (6.67%)	3 (20.00%)	7 (46.67%)	4 (26.67%)	
After 12 weeks	*T. chebula*	5 (33.33%)	6 (40.00%)	3 (20.00%)	1 (6.67%)	0.548
	Hydroquinone	2 (13.33%)	6 (40.00%)	5 (33.33%)	2 (13.33%)	

* Chi-square test

## Discussion

The results of the present study showed the efficacy of *T. chebula* and hydroquinone creams in treating melasma because both creams significantly decreased mMASI scores during the study. However, the onset of action was significantly more rapid with *T. chebula *cream compared to the hydroquinone cream. *T. chebula *cream was significantly effective in reducing mMASI scores after 4 weeks, but this was after 8 weeks for hydroquinone cream. Also, the efficacy of both creams in decreasing mMASI scores did not differ at 4, 8 and 12 weeks after the start of the study.

With reference to some previous clinical studies, some medicinal plants with tyrosinase inhibitory activity like licorice, mulberry and ginseng showed promising efficacy in treating melasma (Nomakhosi and Heidi, 2018). Besides, plants with antioxidant compounds in both oral and topical use showed acceptable efficacy in treating melasma by reducing oxidative stress associated with hyperpigmentary disorders (Babbush et al., 2021). Oral consumption of *T. chebula *was mentioned as a nutritional advice to improve melasma in Iranian traditional medicine (Mojtabaee et al., 2016). In addition, topical application of the plant is traditionally used for treating hyperpigmentation caused by burning. It was reported that *T. chebula* extract at concentrations of 10 to 50 μg/ml had no cytotoxic effects on B16/F10 murine melanoma cells. Also, *T. chebula *could reduce intercellular melanin content about 28.9% at 50 μg/ml concentration (Jamshidzadeh et al., 2017). But until now, no clinical trial has investigated the effect of *T. chebula *on treating melasma. Only one study has evaluated the effect of *T. chebula *5% cream on the skin of 11 healthy, male volunteers for 8 weeks compared to that of the base cream (without *T. chebula *extract). It was found that *T. chebula *5% cream decreased melanin contents of the skin after 8 weeks, but it did not reach statistical significance. This result may be due to the short duration of the study. Also, the cream could decrease erythema and irritation of the skin related to its anti-inflammatory properties. This finding demonstrated that *T. chebula *5 % cream could be safely used without irritating the skin. Furthermore, the cream improved the skin hydration and had anti-wrinkle effect (Akhtar et al., 2012). In the mentioned study, no adverse effects were reported but in the current study, irritation and pruritus were the observed adverse effects in *T. chebula* group.

Flavonoids and phenolic acids (especially gallic acid and ellagic acid) are the main constituents of *T. chebula *fruit that induce tyrosinase inhibitory activity. Also, tyrosinase inhibitory effect of *T. chebula *is correlated to its antioxidant activity (Ansari et al., 2011). Phenolic compounds such as gallic acid, ellagic acid, corilagin, chebulagic acid and chebulinic acid are the major components of *T. chebula* contributing to its antioxidant effect (Bulbul et al., 2022; Suksaeree et al., 2022). In addition, tannins of *T. chebula* like chebulagic acid, chebulinic acid, and corilagin possess anti-inflammatory activity (Bulbul et al., 2022). Moreover, the skin moisturizing property of *T. chebula* fruit may be related to its vitamin C content that increases the biosynthesis of collagen in dermis (Akhtar et al., 2012). 

Although hydroquinone is one of the most common drugs in treating melasma, it has some side effects. Its major side effect is mild skin irritation (Erum et al., 2019). In the present study, *T. chebula* cream was better tolerated. It caused significantly lower irritation in comparison with hydroquinone cream which might be related to *T. chebula *anti-inflammatory effect. Irritation was the most common side effect of hydroquinone cream in this study. The frequent side effects of hydroquinone cream that were previously reported included erythema, irritation, desquamation, burning sensation and pruritus (El‐Husseiny et al., 2020; Janney et al., 2019; Lima et al., 2021). Also, in the current study, desquamation and pruritus in the hydroquinone group and pruritus in* T. chebula *group were observed.

The main limitation of the present study was the small sample size. It is suggested to perform longer duration trials with a larger sample size for both genders to confirm the robustness of the present study results. Another limitation was related to sample size calculation because there was not any similar pilot study. Also, creams with different percentages of *T. chebula *should be prepared and evaluated. Additionally, it is recommended to measure pigment density of patients. Moreover, in the present study, the concentration of hydroquinone in the used hydroquinone cream (2%) was lower than the recommended strength of prescription products (4 or 5%). The reason for this was safety concerns regarding *T. chebula* concentrations more than 5% in the cream (the tyrosinase inhibitory effect of *T. chebula* 5% cream is equivalent to hydroquinone 2% cream).

In conclusion, *T. chebula* 5% cream could be as effective as hydroquinone 2% cream in treating melasma with fewer side effects. It appears to be a promising safe treatment for melasma, but further research is needed to confirm these findings.
